# The transcription factor NF‐YA10 determines the area explored by *Arabidopsis thaliana* roots and directly regulates *LAZY* genes

**DOI:** 10.1111/tpj.70016

**Published:** 2025-03-06

**Authors:** Andana Barrios, Nicolas Gaggion, Natanael Mansilla, Thomas Blein, Céline Sorin, Leandro Lucero, Enzo Ferrante, Martin Crespi, Federico Ariel

**Affiliations:** ^1^ Institute of Plant Sciences Paris Saclay IPS2, CNRS, INRA Université Evry, Université Paris‐Saclay Bâtiment 630 Orsay 91405 France; ^2^ Institute of Plant Sciences Paris‐Saclay IPS2 Université de Paris Bâtiment 630 Orsay 91405 France; ^3^ Instituto de Agrobiotecnología del Litoral, CONICET Universidad Nacional del Litoral Colectora Ruta Nacional 168 km 0 Santa Fe 3000 Argentina; ^4^ APOLO Biotech Santa Fe de la Vera Cruz Santa Fe Argentina; ^5^ Instituto de Fisiología, Biología Molecular y Neurociencias (IFIBYNE) CONICET‐Universidad de Buenos Aires Buenos Aires C1428EHA Argentina; ^6^ Facultad de Bioquímica y Ciencias Biológicas Universidad Nacional del Litoral Santa Fe Argentina; ^7^ Instituto de Ciencias de la Computación CONICET‐Universidad de Buenos Aires Buenos Aires C1428EHA Argentina

**Keywords:** NF‐YA10 transcription factor, root development, gravitropism, *LAZY*, high throughput phenotyping

## Abstract

Root developmental plasticity relies on transcriptional reprogramming, which largely depends on the activity of transcription factors (TFs). NF‐YA2 and NF‐YA10 (nuclear factor A2 and A10) are downregulated by the specific miRNA isoform miR169defg. Here, we analyzed the role of the *Arabidopsis thaliana* TF NF‐YA10 in the regulation of lateral root (LR) development. Plants expressing a version of *NF‐YA10* resistant to miR169 cleavage showed a perturbation in the LR gravitropic response. By extracting several features of root architecture using an improved version of the ChronoRoot deep‐learning‐based phenotyping system, we uncovered that these plants showed a differential angle of LRs over time when compared to Col‐0. Detailed phenotyping of root growth dynamics revealed that NF‐YA10 misregulation modulates the area explored by Arabidopsis roots. Furthermore, we found that NF‐YA10 directly regulates *LAZY* genes, which were previously linked to gravitropism, by targeting their promoter regions. Hence, the TF NF‐YA10 is a new element in the control of LR bending and root system architecture.

## Significance Statement

The NF‐YA10 transcription factor participates in the control of genes involved in the gravitropic response of lateral roots in Arabidopsis and shapes the root system architecture by modulating the area explored in the soil.

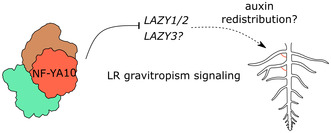

## INTRODUCTION

Plant developmental plasticity relies on a plethora of adaptive strategies in response to the environment. The resulting root system architecture needs to ensure efficient anchor and uptake of water and nutrients. The density and length of lateral roots (LRs) expand the plant surface in contact with the substrate, thus impacting the general growth of the plant (Banda et al., 2019). In the model species *Arabidopsis thaliana*, LR development is tightly regulated by cellular and molecular mechanisms from the very first cell division in the pericycle through the formation of the new meristem and the emergence of the new organ from the main root (MR; Malamy & Benfey, 1997). The intricate regulatory network controlling LR development includes key transcription factors (TFs) integrating internal and environmental cues (Lavenus et al., 2015). The regulatory hub formed by specific miR169 isoforms and their target TF *NF‐YA2* was previously described as a modulator of root architecture. Plants resistant to the miR169‐mediated downregulation of *NF‐YA2* exhibit an enhanced density of LRs explained by altered specific cell type number and greater root meristem size (Sorin et al., 2014). Although the NF‐YA2‐related TF NF‐YA10 (Leyva‐González et al., 2012; Zhang et al., 2017) is also expressed in roots and regulated by miR169, its potential role in LR development remains unexplored. In contrast, NF‐YA10 was found implicated in leaf development, regulating directly IAA biosynthesis (Zhang et al., 2017). In addition, an enhanced stress‐tolerant phenotype was described for plants overexpressing NF‐YA10 and NF‐YA2 (Leyva‐González et al., 2012). A transcriptomic analysis of both transgenic lines hinted at a functional redundancy between both NF‐YA TFs with half of common deregulated genes, in agreement with the high similarity exhibited by all NF‐YA TFs at the protein level (Laloum et al., 2013; Petroni et al., 2012; Siefers et al., 2009). Furthermore, single homozygous mutant lines did not show a major phenotype (Zhao et al., 2020) except for embryo‐lethality in multiple or specific single NF‐YA mutants (Fornari et al., 2013; Pagnussat et al., 2005). In fact, the only *nf‐ya10* mutant available (SALK_126799; Alonso et al., 2003) bears an insertion in the proximal promoter of the gene which resulted in an increase in *NF‐YA10* expression (Figure S1a,c). On the other hand, the reported *nf‐ya2* (GABI44G05; Kleinboelting et al., 2012) mutant bears the insertion in the first intron of the gene, resulting in the knocked‐down expression of a truncated transcript, with higher expression of the 5′ than the 3′ region, downstream the insertion (Figure S1b,d). The activity of the *NF‐YA10* promoter revealed by the control of reporter genes indicated that *NF‐YA10* is expressed in the shoot apical meristem as well as in the MR and LRs and is induced during phosphate deficiency, oppositely to miR169 (Leyva‐González et al., 2012; Sorin et al., 2014). Here, we further characterized NF‐YA10 by undertaking in‐depth characterization of the root system architecture dynamics upon NF‐YA10 deregulation. To this end, we leveraged the potential of ChronoRoot, a high‐throughput automatic phenotyping system based on deep learning (Gaggion et al., 2021), which allows the analysis of several features including standard root metrics (measured every 15 min, then averaged to one value per hour): main root (MR), lateral root (LR), total root (TR) lengths (in mm), number of LRs, LR density: LR length/MR length (mm mm^−1^), discrete LR density: 10 × number of LR/MR length (LRs cm^−1^), ratio of main over total root: MR/TR length, MR, LR, and TR growth speed (mm h^−1^); but also frequency domain metrics: Normalized and detrended MR, LR, and TR growth speed (mm h^−1^), Fourier transform of the growth speeds and convex hull‐related metrics (evaluated at one specific hour per day): convex hull area, width, and height, MR, LR, and TR density over occupied area (mm mm^−2^) and aspect ratio (see “Materials and Methods” section). Here, we expanded the analysis by introducing two new angle measurements as additional features. Then, we used transgenic plants carrying an *NF‐YA10* gene (own promoter and coding region) without the 3′UTR region targeted by miR169 to assess their root architecture. The resulting *NF‐YA10* mRNA is resistant to miR169‐mediated cleavage (NF‐YA10 miRres). These plants exhibit an increased root area when compared to Col0, due not only to a greater LR density but also as a result of an alteration of LR angles, suggesting a link between NF‐YA10 action and gravitropic responses. Furthermore, we demonstrated that NF‐YA10 directly regulates *LAZY* genes, previously linked to the gravitropic response of roots (Kawamoto & Morita, 2022), indicating that NF‐YA10 may act as a coordinator of LR distribution in the rhizosphere, shaping the final surface of the root architecture.

## RESULTS

**Figure 1:**
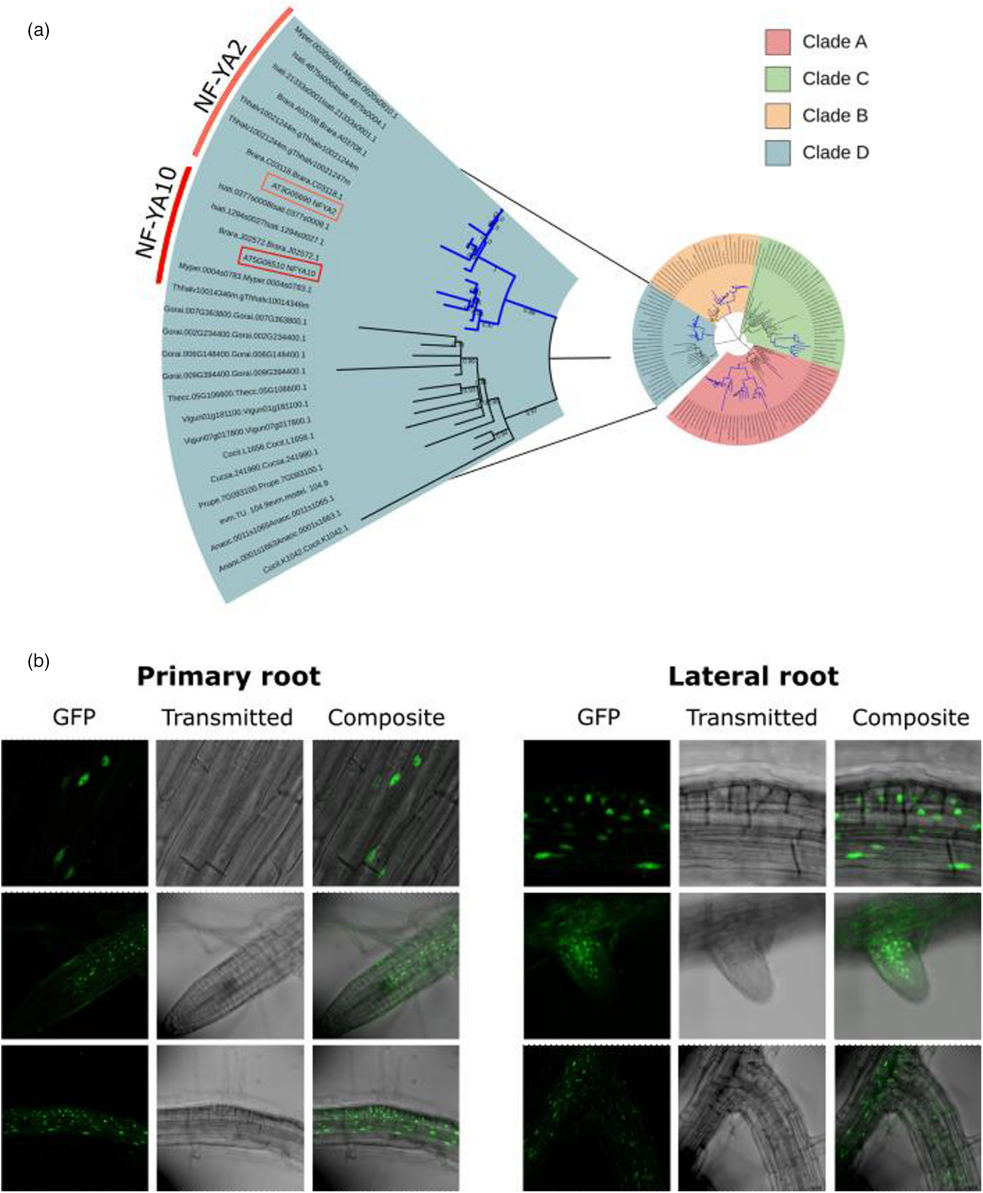
NF‐YA10 diverged from a recent duplication with NF‐YA2 within Brassicaceae and is expressed in nuclei of primary and lateral root vasculature cells. (a) Phylogenetic tree of NF‐YAs with an extended set of Malvidae and Brassicaceae species. *AtNF‐YA10* is highlighted in red and *AtNF‐YA2* in orange. The duplications in Brassicaceae are colored in blue. Only branches with bootstrap values higher than 65% are shown. (b) Localization of NF‐YA10 fused to GFP in primary (left) and lateral (right) roots of *pNF‐YA10:GFP‐NF‐YAmiRres.1* 8‐day‐old plants.

A phylogenetic analysis of plant NF‐YA TFs indicated that this family of proteins presents four clades with internal duplications that are characteristic to the plant kingdom (Figure S2; Table S1). As previously proposed (Laloum et al., 2013), NF‐YA2 and NF‐YA10 (clade D) arose from a recent duplication which seems to be specific to Brassicaceae, similarly to other NF‐YAs such as NF‐YA1/9 (clade C) and NF‐YA4/7 (clade B). NF‐YA6 and NF‐YA5, NF‐YA3, and NF‐YA8 emerged from two duplications inside the clade A (Figure 1a; Table S1). Expression studies based on transcriptional reporter *pNF‐YA10:GUS* assays were used to show *NF‐YA10*‐specific expression in the root vasculature (Leyva‐González et al., 2012; Sorin et al., 2014). In this study, we used transgenic plants expressing the translational fusion *pNF‐YA10:GFP‐NF‐YA10mut* (miR169‐resistant *NF‐YA10* mRNA, called hereafter NF‐YA10 miRres) to localize the NF‐YA10 protein expression pattern. We observed GFP:NF‐YA10 accumulation in the nuclei of root vasculature, endodermis, and cortex cells, and during LR development, from early stages and along the growth of the emerged LR (Figure 1b; Figure S3), suggesting a regulatory role in the development of this organ.

**Figure 2:**
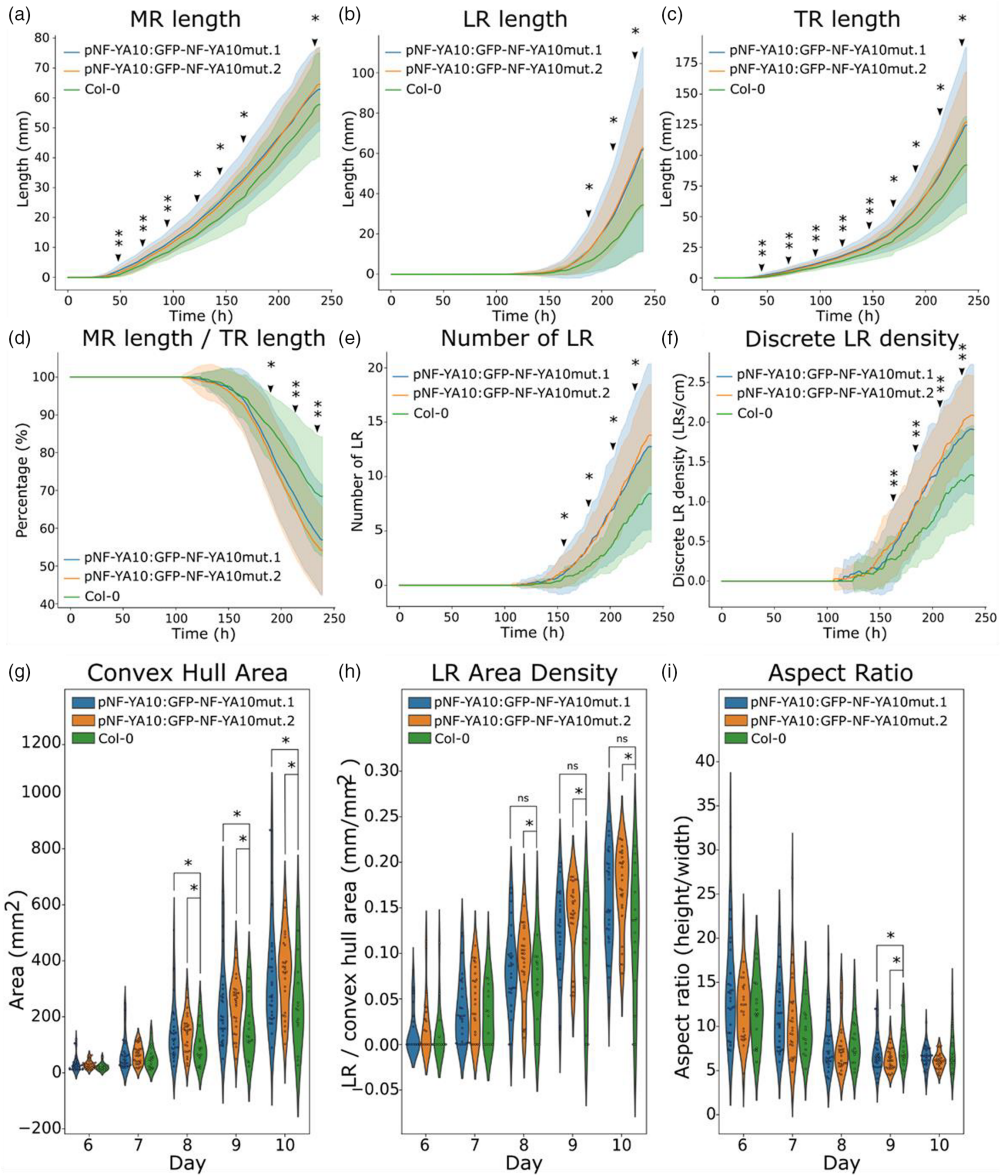
High expression of NF‐YA10 affects root growth and the resulting root system architecture. ChronoRoot measurements of NF‐YA10 miRres and Col‐0 WT plants: main root length (a), lateral root length (b), total root length (c), ratio main root length/total root length (d), number of lateral roots (e), discrete lateral root density (or simply LR density, the term Discrete is used to differentiate the number of LRs from the area occupied, as shown in panel g) (f). Convex hull area (g), lateral root area density (h) and ratio of root system height and width (i) of NF‐YA10 miRres lines and Col‐0 at different ages of the plant. (a–f) Solid lines represent the average value and the bands represent the standard deviation. Asterisks (*) correspond to *P*‐value <0.05 (Mann–Whitney test); in (a–f), “**” mean significance for both NF‐YA10 miRres lines compared to Col‐0. Statistical analysis was performed with *n* ≥ 14 plants for Col‐0 and *n* ≥ 28 and *n* ≥ 24 respectively for *pNF‐YA10:GFP‐NF‐YA10mut.1* and 0.*2* lines.

A comprehensive characterization of the root architecture dynamics was then undertaken by using ChronoRoot (Gaggion et al., 2021), i.e., comparing two independent NF‐YA10 miRres lines and the WT. NF‐YA10 miRres plants showed a slightly longer MR than the WT, a feature observed since the germination (Figure 2a). Interestingly, total LR length in NF‐YA10 miRres plants increased at a greater speed than the WT (Figure 2b). Therefore, although NF‐YA10 miRres plants exhibit a general faster growth of the whole root system (Figure 2c), the relative contribution of the MR to the global root system is less significant (Figure 2d). Moreover, the number of LRs was significantly higher in NF‐YA10 miRres plants (Figure 2e), resulting in an enhanced LR density (Figure 2f). The final root architecture of NF‐YA10 miRres plants exhibited an enhanced covered surface, which is illustrated by the significantly expanded convex hull area of the full root system (Figure 2g). However, the density of the LRs covering the convex hull area (Figure 2h) did not emerge as a distinct feature between both independent lines, whereas the aspect ratio (height/width of the root system) of NF‐YA10 miRres plants was significantly different from the WT, at least at day 9 (Figure 2i), indicating that longer LRs expand away from the MR instead of growing close to the MR axis.

**Figure 3:**
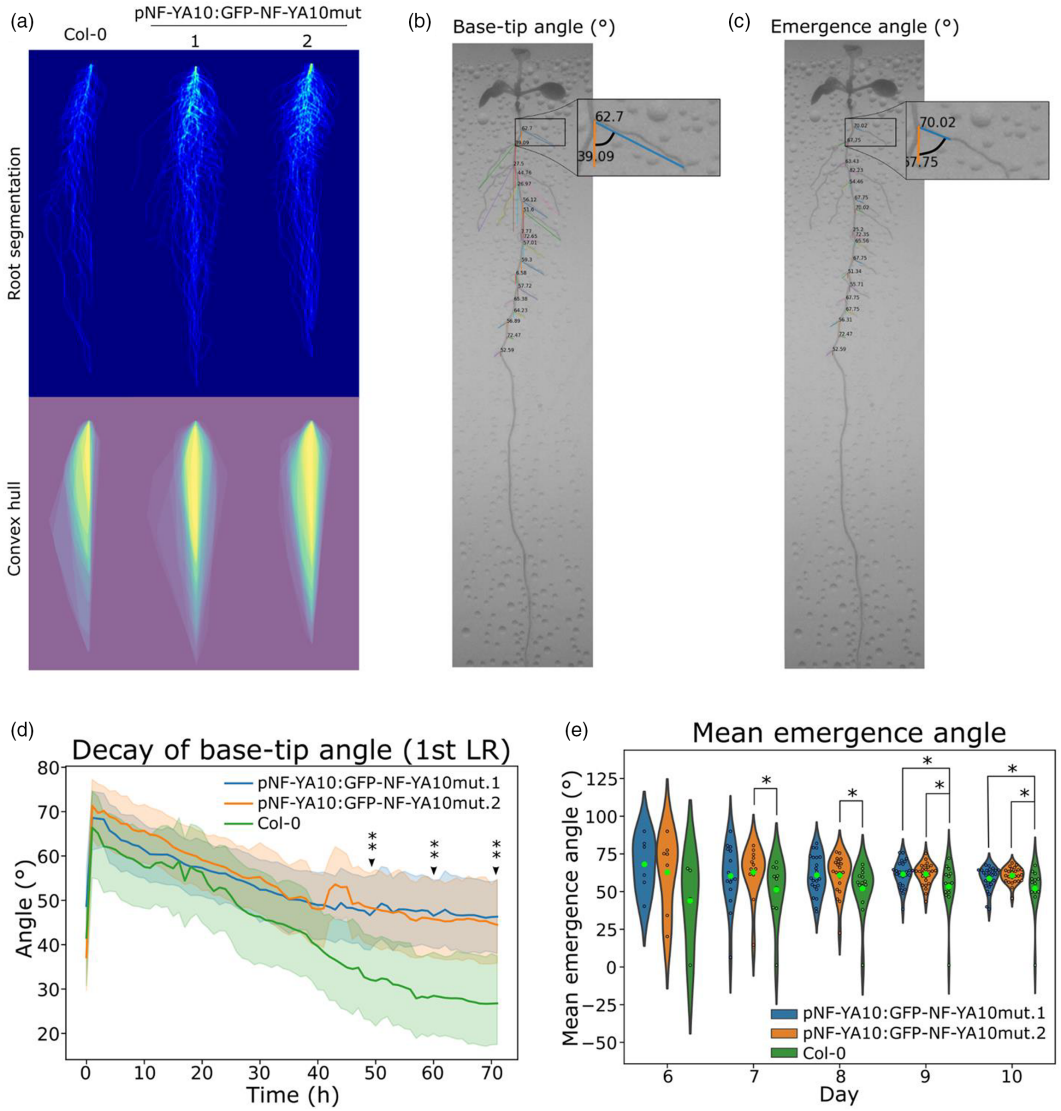
NF‐YA10 is a regulator of lateral root curvature bending shaping the root system architecture. (a) Pictures of NF‐YA10 miRres and Col‐0 plants at day 7 and 10. Superposition of all NF‐YA10 miRres and Col‐0 root profiles in mock conditions. Plant root superpositions are generated by first rotating them to obtain a straight vertical line between the beginning and final point of the MR. They are indeed the real plant roots, but the segmentations not images. The superposition is made by adding them up one on top of each other, alineated by this straight line and putting the MR beginning point at the same pixel. As each segmentation image has a value of 1 in the pixel where the root is present, the color scale is straightforwardly the number of plants in that specific pixel that are overlapped. Representative plant of NF‐YA10 miRres and Zoom on a representative plant of NF‐YA10 miRres.1 for the annotation of the two novel ChronoRoot measurements: (b) base‐tip angle and (c) emergence angle respectively. (d) Dynamics of tip decay of the first lateral root to emerge along the time of NF‐YA10 miRres plants and Col‐0. The base‐tip angle was measured, for Col‐0, on *n* ≥ 14 roots and for *pNF‐YA10:GFP‐NF‐YA10mut.1* and 0.*2* lines, *n* ≥ 28 and *n* ≥ 24 respectively. (e) Mean emergence angle of NF‐YA10 miRres and Col‐0 roots at different ages of the plant. Asterisks (*) correspond to *P*‐value <0.05 (Mann–Whitney test). (**) mean significance for both NF‐YA10 miRres lines compared to Col‐0. The emergence angle was measured on *n* ≥ 10 roots for Col‐0 and *n* ≥ 16 and *n* ≥ 15 respectively for *pNF‐YA10:GFP‐NF‐YA10mut.1* and 0.*2* lines.

This characteristic feature led us to wonder if the angle of the LRs was affected in NF‐YA10 miRres plants. To address this issue, two additional parameters were incorporated into ChronoRoot by leveraging information from both the segmentation and graph structures generated by the deep learning‐based model. The bending of LRs is reliant on the gravitational perception by the columella cells, in conjunction with endogenous signals. This interplay accounts for the observed bending preceding the amyloplast formation (Guyomarc'h et al., 2012). The first parameter now determined by ChronoRoot is the angle of emergence between the initial millimeter of the LR and the MR, determined as described by Guyomarc'h et al. (2012). This parameter reflects the integration of developmental cues occurring prior to the perception of gravity, mediated by the relocalization of amyloplasts. In contrast, the second parameter, termed the base‐tip angle (measured from the base to the tip of each LR), exhibits a gradual decline, highlighting the predominant influence of gravity on LR development. Both angles are quantified relative to the gravity vector. The combination of these two parameters offers valuable insights into the developmental transition occurring in LRs after emergence, influenced by the force of gravity. These parameters allowed for tracking the evolution of the base‐tip angle and the emergence angles of LRs over time (Figure 3a–d; Figure S4a; Table S2), thereby expanding the original ChronoRoot applications. Considering the first LR emerged per plant, NF‐YA10 miRres seedlings present a higher base‐tip angle than the WT during the emergence of LR, reaching a 22°‐difference after 3 days (Figure 3e). Accordingly, the emergence angle of LR was also affected, as NF‐YA10 miRres plants showed a trend of greater emergence angles than the WT starting from the emergence of the first LR (day 6), which was statistically significant for both independent lines from day 9 on (Figure 3f). Although the emergence angle does not change over time, it is worth noting that since day 9 enough LRs have emerged to make the difference statistically relevant. Notably, the higher density of the LR area observed in NF‐YA10 mirRes.2 seedlings seems related to the higher discrete LR density (the number of LRs cm^−1^) and the sum of the length of LRs; whereas the convex hull area and the aspect ratio are significantly affected in both independent lines as the result of the different LR bending exhibited by NF‐YA10 miRes plants.

Considering that NF‐YA10 and NF‐YA2 miRres plants exhibited similar root developmental phenotypes (Sorin et al., 2014; Figure 2), we also assessed the LR bending of NF‐YA2 miRres plants, which exhibited a delayed bending compared to WT, similarly to NF‐YA10 miRres plants (Figure S4b,c). The potential role of NF‐YA10 in gravitropism was also assessed in the MR growth during a bending assay (i.e., plant plates turned 90° and MR growing angles measured afterward). Interestingly, one of the two NF‐YA10 miRres lines showed a significantly delayed response compared to WT (Figure S5).

**Figure 4:**
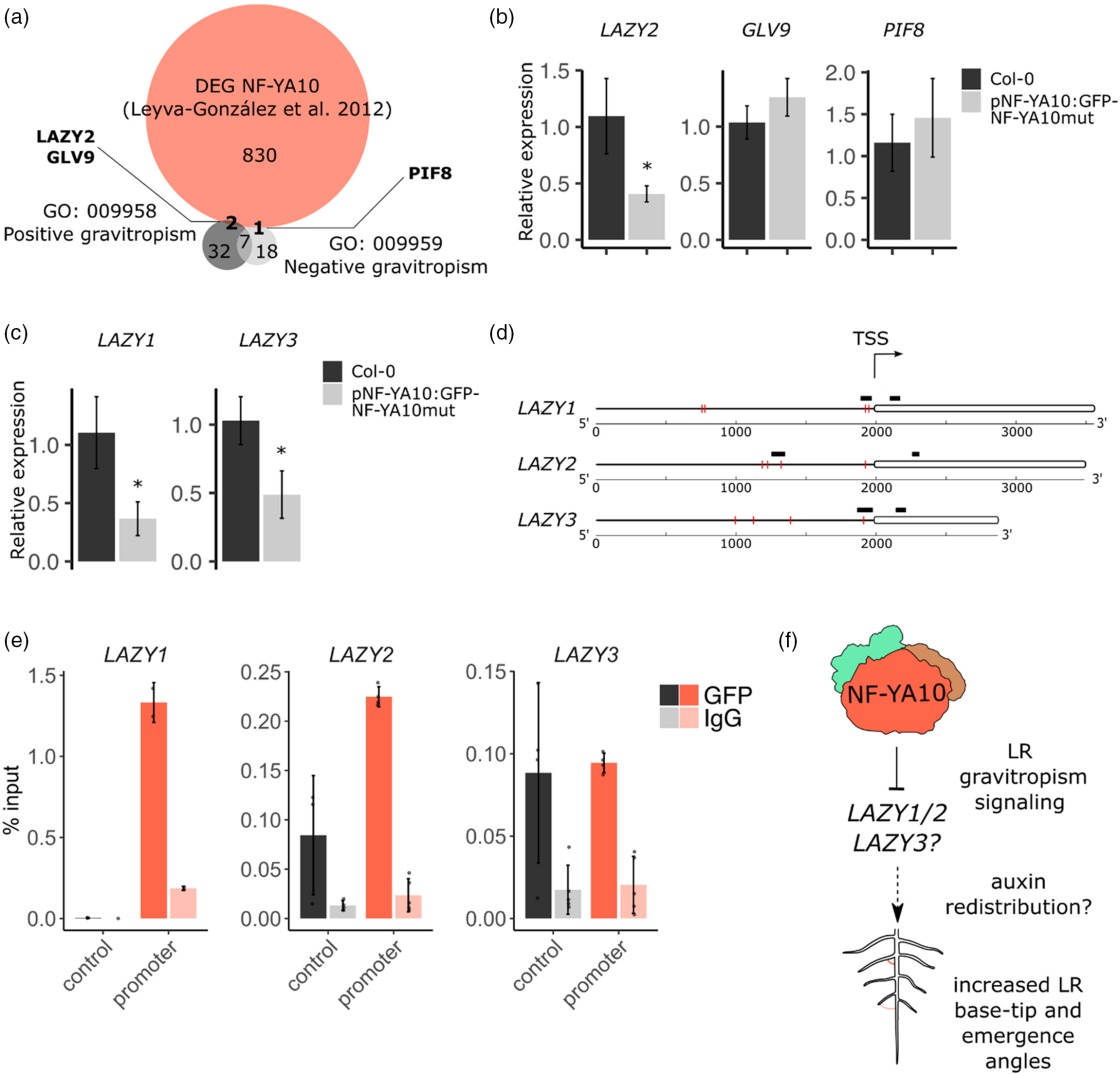
NF‐YA10 binds *LAZYs* promoters, resulting in transcriptional repression of these genes. (a) Venn diagram of DEGs from Leyva‐González et al. (2012) and genes comprised in Gene Ontology terms of positive and negative gravitropism. (b) Location of CCAAT boxes are indicated in red lines and the respective DNA regions assessed in ChIP‐qPCR in black for each of the genes *LAZY1*, *LAZY2* and *LAZY3*. (c) Relative expression of LAZYs in NF‐YA10 miRres and Col‐0 roots of 8‐day‐old seedlings. (d) Schematic representation of the regions assessed for the experiment shown in the following panel. (e) Chromatin immunoprecipitation (ChIP)‐qPCR analysis of NF‐YA10 binding at *LAZYs* gene and promoter regions in 8‐day‐old NF‐YA10 miRres and Col‐0 seedlings. (f) Proposed model for the NF‐YA10‐dependent lateral root gravitropic response. NF‐YA10 participates in NF‐Y trimer TF complexes, directly activating or repressing a subset of target genes involved in LR bending and gravitropism. In (b, c, e), values correspond to the mean and error bars to the standard deviation of three biological replicates. The asterisks (*) indicate a *P*‐value <0.05 (Mann–Whitney test).

To decipher the molecular mechanism behind this phenotype, we looked for potential NF‐YA10 targets involved in lateral organ gravitropism described in the literature, which bear CCAAT‐boxes in their promoter sequences. To this end, we first crossed the list of differentially expressed genes (DEGs) in NF‐YA10‐inducible overexpressing seedlings (Leyva‐González et al., 2012) with TAIR Gene Ontology lists for genes related positively or negatively to gravitropism (Table S3). Among them, we identified the genes *LAZY2* and *GLV9*, and *PIF8*, respectively (Figure 4a). In contrast to *GLV9* and *PIF8*, *LAZY2* was transcriptionally deregulated in roots of NF‐YA10 miRres plants (Figure 4b). Considering the behavior of *LAZY2* in NF‐YA10‐deregulated plants, we further investigated the transcriptional levels of members of the LAZY protein‐encoding gene family, which have been linked to redundant activity (Jiao et al., 2021; Taniguchi et al., 2017). Particularly, *LAZY1/2/3* are known to be essential for accurate gravitropic auxin‐driven sensing of lateral and primary roots and shoots (Furutani et al., 2020; Taniguchi et al., 2017; Waite & Dardick, 2024). Interestingly, it was shown that LRs in the *lazy1* and *lazy1/lazy2/lazy3* triple mutant display a disturbed gravitropic response (Hollender et al., 2020; Jiao et al., 2021; Taniguchi et al., 2017). Thus, we assessed their level of expression in NF‐YA10 miRres roots. *LAZY1*, *LAZY2*, and *LAZY3* were all repressed compared to WT (Figure 4c), in agreement with the gravitropic phenotypes observed in the *lazy* mutants. In order to determine if NF‐YA10 directly regulates these genes, we performed ChIP‐qPCR targeting TSS‐proximal CCAAT boxes present in their promoter regions compared to ChIP enrichment in their respective gene bodies taken as the negative control (qPCR probes distribution indicated in Figure 4d). In basal conditions, *LAZY1* and *LAZY2* emerged as direct targets of NF‐YA10 (Figure 4e). *LAZY3* appears only as a potential direct target, considering that the negative control within the gene body is close to the CCAAT box assessed. Altogether, our results suggest that regulation of NF‐YA10 expression is a new element involved in the control of the root system architecture, notably determining the final volumetric root distribution in the soil by modulating root growth, LR development, and their gravitropic response. NF‐YA10 recognizes the promoters and regulates a subset of root developmental genes, including *LAZY* genes, known to be involved in LR gravitropic signaling suggesting that it may be a new regulatory circuit controlling the long‐term surface of the root system (Figure 4f).

## DISCUSSION

In animals, the NF‐Y complex is described as a pioneer TF (Oldfield et al., 2014), able to recruit chromatin remodelers and downstream TFs to modulate the activity of target genes. In Arabidopsis, NF‐YA, NF‐B and NF‐YC are encoded by 10, 13 and 13 genes, respectively, boosting the diversification of molecular and physiological roles of this TF family, based on the wide range of combinations likely occurring in different cell types from the organism (Laloum et al., 2013). Among them, NF‐YA2 and NF‐YA10 emerged from a recent Brassicaceae‐specific duplication inside a clade with low divergence degree, thus exhibiting few changes in the protein sequence. *NF‐YA2* and *NF‐YA10* are targets of the specific isoform of miR169‐defg, and plants with a NF‐YA10 gene copy resistant to the downregulation driven by this miRNA showed similar LR phenotype to NF‐YA2 miR‐resistant lines (Sorin et al., 2014; Figure 2; Figure S4b,c). For a better characterization of NF‐YA10 miRres plants, two additional root parameters were determined by ChronoRoot through the development of ad hoc deep segmentation networks: base‐tip angle and emergence angle of LRs. These measures were 22° (day 6) and 17° (60 h after germination) higher in NF‐YA10 miRres LRs than in the WT, causing, together with higher LR density, a significant increase of the area occupied by the root system in two dimensions over the agar plate (Figure 3; Figure S4a; Table S2). Strikingly, Arabidopsis WT roots exhibit a similar phenotype in response to phosphate starvation (Bai et al., 2013) and it was previously shown that the promoter of *NF‐YA10* is induced under these conditions, whereas miR169 was repressed (Leyva‐González et al., 2012). Notably, the miR‐resistant version of *NF‐YA2*, the closest gene related to *NF‐YA10*, also exhibited an altered LR bending, suggesting that these recently diverged genes may exert overlapping roles in LR development. Considering that root‐specific targets of NF‐YA10 are yet unknown, we searched for potential targets that could be linked to the regulation of LR gravitropism, among which we identified the *LAZYs* genes, described as components of a signaling pathway controlling gravitropism in shoots and roots (Chen et al., 2023; Furutani et al., 2020; Kawamoto & Morita, 2022; Taniguchi et al., 2017; Waite & Dardick, 2024). All three genes assessed turned out to be downregulated in NF‐YA10 miRres plants. ChIP‐qPCR showed that *LAZY1* and *LAZY2* promoters are bound by NF‐YA10, in contrast to *LAZY3* when compared to the nearby negative control. Further research will be required to determine if NF‐YA10 may participate in the control of LR gravitropism in response to low phosphate concentrations.

Notably, lugol‐staining of LRs revealed no observable changes in amyloplast distribution in NF‐YA10 miRres roots (Figure S6), suggesting that NF‐YA10 may mediate developmental signals affecting the gravitropic response rather than the capacity of the LR to sense gravity through the columella cells. It was shown that LR orientation depends on gravity sensing by the columella cells, as well on endogenous signals, explaining why the bending is observed prior to the formation of amyloplasts (Guyomarc'h et al., 2012). Interestingly, an analysis conducted on public root single‐cell RNA‐seq data (Shahan et al., 2022) revealed that *NF‐YA10* exhibited low expression levels, predominantly within a limited number of cells of xylem, procambium, pericycle, and endodermis but not in columella cells (Figure S7). *LAZY1* and, to a lesser extent, *LAZY2/3* displayed an expression pattern that includes endodermal cells, indicating that NF‐YA10 may control these genes within this cell type. Although *LAZY2* and *3* are highly expressed in gravity‐sensing columella cells, the expression of *LAZY* genes in other cell types expands their potential role in the integration of developmental signals, notably in the endodermis. Several studies have shown that the endodermis plays an essential role in the progression to the LR initiation stage in the neighboring founder cells (Marhavý et al., 2013; Vermeer et al., 2014). In addition, recent studies suggest that the endodermis acts as a barrier and controls the emergence of LRs (Seo et al., 2021). Furthermore, statolith accumulation is influenced by sugar transport, with the functionality of vascular tissue likely playing a crucial role in determining the sink capacity of the LR apex and the columella's amyloplast deposition. Very recently, LAZY proteins were discovered to accumulate at the periphery of amyloplasts and repolarize in response to gravity in the back of statoliths, where they are MPK‐phosphorylated (Chen et al., 2023). LAZY proteins thus participate in the redistribution of auxin flow through the recruitment of cofactors and PIN3 relocalization (Furutani et al., 2020). Overall, auxin fulfills multiple roles throughout LR development from initiation to emergence (Du & Scheres, 2018). Interestingly, lower levels of auxin were detected in NF‐YA10 overexpressing leaves, and a microarray transcriptomic approach of the same plants served to determine that the expression of a significant subset of auxin‐related genes depends on NF‐YA10 (Zhang et al., 2017). This further suggests that this TF may play an important role in auxin homeostasis, likely explaining the phenotypes observed in root growth, development and LR gravitropism. Furthermore, the use of the synthetic reporter DR5 allowed demonstrating that auxin signaling was increased in triple *lazy2/lazy3/lazy4* mutant roots (Ge & Chen, 2019; Yoshihara & Spalding, 2017), further linking the impact of the NF‐YA10‐LAZYs hub on auxin‐driven root development. In addition, the ortholog of Arabidopsis *LAZY1* in maize, ZmLAZY1, directly interacts with the early auxin response factor ZmIAA17 in the nucleus of maize cells (Dong et al., 2013). In Arabidopsis, the dwarf late‐senescent phenotype of NF‐YA10 overexpressing plants under the control of a 35S promoter was previously proposed to be caused by a reduction of plant growth directed by transcriptional regulation of cell wall and sucrose pathways actors (Leyva‐González et al., 2012). In contrast to 35S‐mediated overexpression, a miR‐resistant version induces a slight upregulation and in the same cells expressing the NF‐YA10 TF. Therefore, we propose that NF‐YA10 contributes to orchestrate LR development in addition to auxin and cell expansion related genes by fine‐tuning gravitropism signaling through regulation of *LAZY* genes expression. It was previously reported that the promoter of *LAZY1* was only active in aerial tissues, and the *lzy1* phenotype is mainly related to the shoot branch angle (Taniguchi et al., 2017). However, here we showed that *LAZY1* transcripts are specifically detected in root endodermal cells, as revealed by single‐cell RNA‐Seq (Figure S7). Considering that no shoot branching phenotype was observed in NF‐YA10 mirRes plants, we propose that the regulation of NF‐YA10 over *LAZY* genes may occur predominantly in roots. Indeed, NF‐YA10 deregulation results in a larger root area. It was shown that an enhanced root surface area positively correlated with plant weight, opening new perspectives about the potential of NF‐Y TFs for the improvement of relevant traits for hydroponic cultures and more globally for agriculture (Yang et al., 2021). Further research about how LRs explore the substrate under the control of transcriptional regulators could help to improve crop growth in response to nutrient limitations, including phosphates.

## MATERIALS AND METHODS

### Plant lines generated and used for this study

All plants used in this study are in Columbia‐0 background. pNF‐YA10:GFP‐NF‐YA10miRres lines were obtained using the GreenGate vectors (Lampropoulos et al., 2013), using 2000‐pb region upstream of the start codon of *NF‐YA10* amplified from genomic DNA and CDS of NF‐YA10 without miR cleavage site amplified from cDNA. Arabidopsis plants were transformed by floral dip (Clough & Bent, 1998) using *Agrobacterium tumefaciens* C58. *nf‐ya2* (GABI44G05; Kleinboelting et al., 2012) and *nf‐ya10* (SALK_126799; Alonso et al., 2003) described in Figure S1 were ordered from NASC. The homozygosity NF‐YA10 miR169‐resistant lines and T‐DNA mutants were identified by PCR (see primers in Table S4).

### Growth conditions and phenotypic analyses

For phenotype analyses, plants were grown vertically on plates placed in a growing chamber in long day conditions (16 h in light 130 μE; 8 h in dark; 23°C). Plants were grown on solid half‐strength MS medium (MS/2) supplemented with 0.8 g L^−1^ agar (Sigma‐Aldrich, St. Louis, USA; A1296 #BCBL6182V) and 1% sucrose, buffered at pH 5.6. Temporal phenotyping was performed using ChronoRoot (Gaggion et al., 2021). In the case of gravitropic assays, plates were rotated at 90° when reaching 7 DAS and photographed after 24 h. Images were then analyzed with ImageJ to measure the root tip angle of each plant (*n* = 15) formed after reorientation. For the observation of amyloplasts, 8‐day‐old plants (*n* = 15) were dipped for 8 min in Lugol staining solution (0.1% I, 1% KI; Sigma‐Aldrich) and observed in an Eclipse E200 Microscope (Nikon) equipped with a Nikon D5300 camera.

### Confocal laser scanning and fluorescence microscopy (CLSM)

For CLSM, roots of stable two independent lines of NF‐YA10 miRres plants were imaged with a Leica TCS SP8 confocal laser scanning microscope. For GFP signal imaging, samples were excited at 488 nm, the detection was set at 493–530 nm, and the transmitted light was also imaged. All the images were captured using a 20× or 63× lens. Image processing was performed using Fiji software (Schindelin et al., 2012).

### Sequence alignment and phylogenetic tree analysis

Protein sequences corresponding to plant NF‐YA family members were identified using the BLASTP tool (Boratyn et al., 2013) and downloaded from Phytozome 13 (https://phytozome‐next.jgi.doe.gov/) (Goodstein et al., 2012). Proteins from other organisms were obtained from the NCBI database. For the tree in Figure S1, protein sequences of plant species were selected by choosing members from the main phylogenetic clades, giving a total of 81 protein sequences (Table S1). Three no‐plant species sequences were used for tree rooting. For the tree in Figure 1(a), several protein sequences from Brassicaceae and Brassicales‐Malvales species were added to improve resolution in this group of organisms, resulting in a total of 137 protein sequences (Table S1). The alignments were made using MUSCLE default parameters (Edgar, 2004). The phylogenetic trees were built using the Seaview 4.5.0 software and the PhyML‐aLRT‐SH‐LIKE algorithm (Gouy et al., 2010) with maximum likelihood tree reconstruction. A model of the amino acid substitution matrix was chosen through the Datamonkey bioinformatic server (www.datamonkey.org; Delport et al., 2010), which selected the VT model. The resulting trees were represented using iTOL (http://itol.embl.de/itol.cgi; Letunic & Bork, 2016), showing branches with bootstrap values higher than 65%.

### Quantification of transcript levels by RT‐qPCR

Total RNA was extracted from roots using TRI Reagent (Sigma‐Aldrich) and treated with DNaseI (NEB) as indicated by the manufacturers. Reverse transcription was performed using 1 μg total RNA and the M‐MLV reverse transcriptase (Promega). qPCR was performed on a StepOne™ Real‐Time PCR System (Thermo Fisher) with Sso Advanced Universal mix (BioRad) in standard protocol (40 cycles, 60°C annealing). Primers used in this study are listed in Table S4. Data were analyzed using the ΔΔ*C*_t_ method using ACTIN (AT3G18780) for gene normalization (Czechowski et al., 2005).

### Chromatin immunoprecipitation

Three biological replicates of 8 DAS whole seedlings grown in control condition were collected. ChIP was performed using anti‐GFP (Abcam; ab290) and anti‐immunoglobulin G (IgG) anti‐IgG (Abcam; ab6702), as described in Ariel et al. (2020), starting from 5 g of seedlings crosslinked in 1% (v/v) formaldehyde. Chromatin was sonicated in a water bath Bioruptor Plus (Diagenode; 10 cycles of 30 sec ON and 30 sec OFF pulses at high intensity). Antibody‐coated Protein A Dynabeads (Invitrogen) were incubated 12 h at 4°C with the samples. Immunoprecipitated chromatin was reverse‐crosslinked with 20 mg of Proteinase K (Thermo Fisher; #EO0491) overnight at 65°C. Finally, DNA was recovered using phenol/chloroform/isoamyl acid (25:24:1; Sigma) followed by ethanol precipitation. For input samples, 10% of sonicated chromatin was collected for each sample before the immunoprecipitation and reverse‐crosslinked and extracted as the immunoprecipitated samples. Results are expressed as enrichments, corresponding to GFP or IgG percent of input, measured by qPCR (primers used are listed in Table S4).

### Construction of angle parameters based on ChronoRoot

To construct the new angle parameters, specifically the base‐tip angle (Figure 3b) and the emergence angle of LR (Figure 3c), we first proceeded on extracting the LRs from the labeled skeleton of each plant. Then, to preserve the labels and information pertaining to the order in which the LRs began to grow, we matched the extracted LRs across time‐steps. We performed the matching from one time‐step to the previous one by using the pixel position where the root initiated. With the position, skeleton, and label of each plant root established, we proceeded to measure the LR base‐tip angle. This angle was calculated for each LR by examining the right triangle formed by the base position, the tip, and the *Y*‐axis. For the second angle parameter, i.e., the emergence angle, instead of measuring it from the base to the tip, we established a fixed distance in millimeters (2 mm in our study, though adjustable as a parameter) to construct the right triangle (see Figure 3c). We traversed 2 mm along the skeleton from the base and measured the angle between the *Y*‐axis and this point to calculate it. Source code for ChronoRoot is publicly available on GitHub (https://github.com/ngaggion/ChronoRoot, Gaggion et al., 2021).

## CONFLICT OF INTEREST

The authors declare that they have no competing interests.

## Supporting information


**Figure S1.** Characterization of *nf‐ya2* and *nf‐ya10* insertional mutants.
**Figure S2.** Phylogenetic tree of NF‐YAs in plant and non‐plant organisms.
**Figure S3.** Localization of NF‐YA10‐GFP during lateral root development.
**Figure S4.** Analysis of novel lateral root curvature parameters in NF‐YA2miRres and NF‐YA10miRres seedlings using ChronoRoot.
**Figure S5.** Characterization of the gravitropic response of the main roots of NF‐YA10.miRres seedlings.
**Figure S6.** Characterization of amyloplasts NF‐YA10 miRres main and lateral root tips.
**Figure S7.** *NF‐YA10* and *LAZY* genes are expressed in multiple cell types according to single‐cell transcriptomics in Arabidopsis roots.

**Table S1.** Table of species and sequences used for phylogenetic analyses.

**Table S2.** Measures of emergence and base‐tip mean angles of lateral roots for Col‐0 and NF‐YA10 miRres genotypes.

**Table S3.** Lists of differentially expressed genes (DEGs) in *NF‐YA10*‐inducible overexpressor (Leyva‐González et al., 2012) and genes assigned to TAIR Gene Ontology terms related positive and negative gravitropism.

**Table S4.** Table of primers used in this study.

## Data Availability

All relevant data can be found within the manuscript and its supporting materials.
