# Supplementary material for: The transcription factor NF‐YA10 determines the area explored by Arabidopsis thaliana roots and directly regulates LAZY genes
Source: Plant J. 2025 Mar 6;121(5):e70016. doi: 10.1111/tpj.70016 (PMC11885863; doi:10.1111/tpj.70016)
Supplement: Supplementary file 1 — Figure S1. Characterization of nf‐ya2 and nf‐ya10 insertional mutants. Figure S2. Phylogenetic tree of NF‐YAs in plant and non‐plant organisms. Figure S3. Localization of NF‐YA10‐GFP during lateral root development. Figure S4. Analysis of novel lateral root curvature parameters in NF‐YA2miRres and NF‐YA10miRres seedlings using ChronoRoot. Figure S5. Characterization of the gravitropic response of the main roots of NF‐YA10.miRres seedlings. Figure S6. Characterization of amyloplasts NF‐YA10 miRres main and lateral root tips. Figure S7. NF‐YA10 and LAZY genes are expressed in multiple cell types according to single‐cell transcriptomics in Arabidopsis roots. [file TPJ-121-0-s002.docx]

**SUPPLEMENTAL FIGURES**


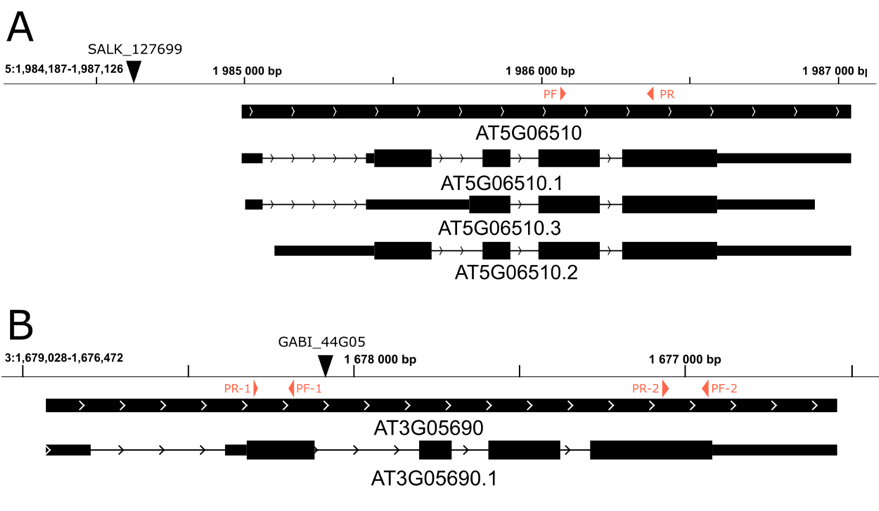


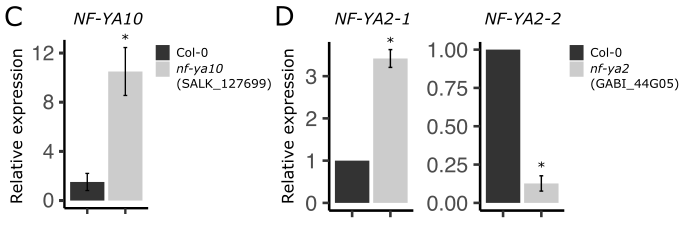


**

**

**Figure S1. Characterization of *nf-ya2* and *nf-ya10* insertional mutants.** (A) Genomic localization of *NF-YA10*: the site of T-DNA insertion (SALK_126799) is indicated by a black arrow and RT-qPCR primers are represented in orange. (B) Genomic localization of *NF-YA2*: the site of T-DNA insertion (GABI_44G05) is indicated by a black arrow and RT-qPCR primers are represented in orange. (C) *NF-YA10* expression in *nf-ya10* mutant (SALK_126799) and Col-0 seedlings. (D) *NF-YA2* expression in *nf-ya2* mutant (GABI_44G05) and Col-0 seedlings. *NF-YA2-1* and *NF-YA2-2* correspond to expression levels measured using respectively primers PR/PF-1 and PR/PF-2 from Figure S1B. (E) Root architecture characterization of 7-day-old nf-ya2 mutant seedlings using RootNav (Pound et al., 2013). No significant differences were observed in any assessed parameter between genotypes. Statistical analysis was performed on n=29 plants for each genotype, using the Welch t-test.

**
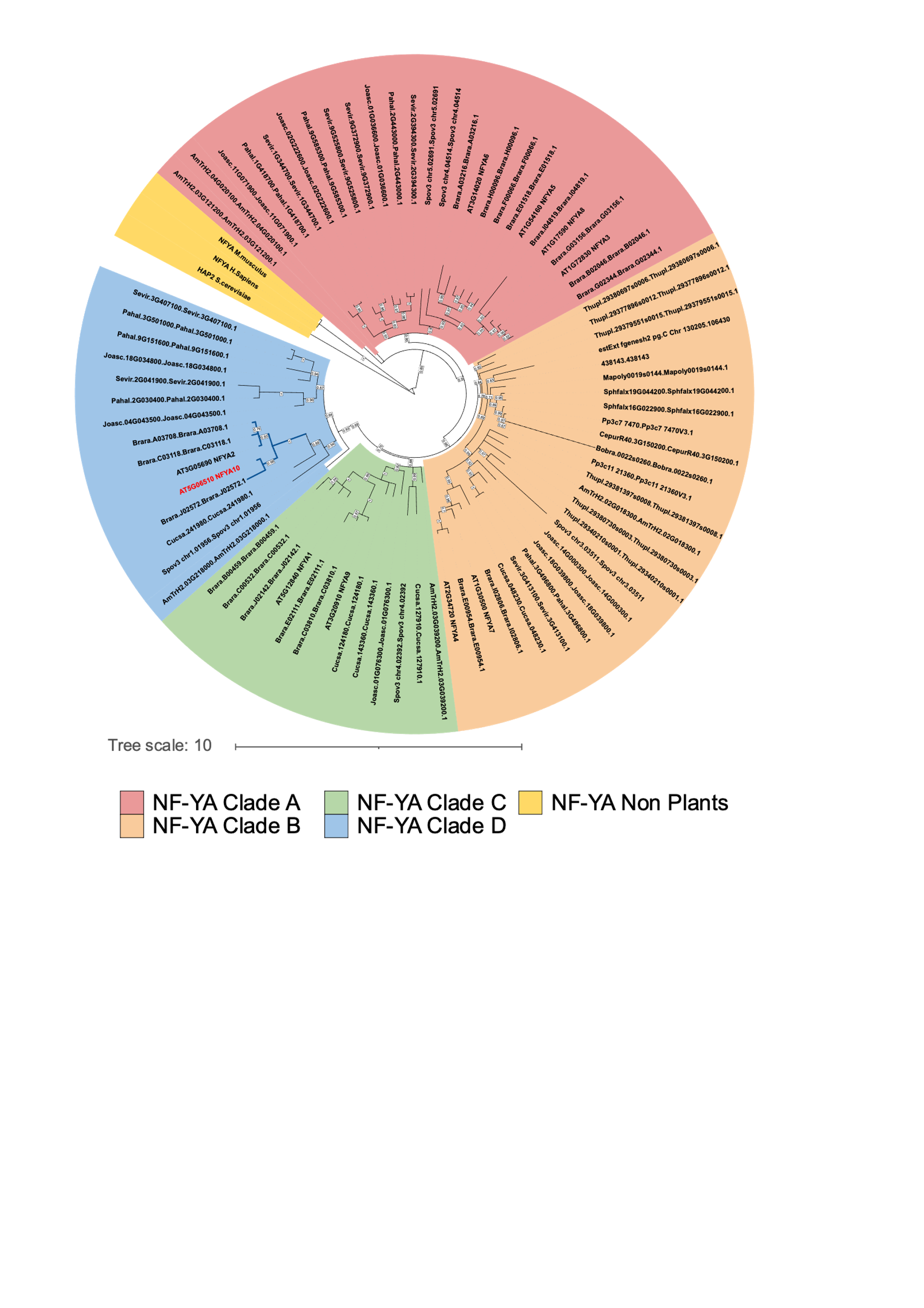
Figure S2.** **Phylogenetic tree of NF-YAs in plant and non-plant organisms**. *AtNF-YA10* is highlighted in red.


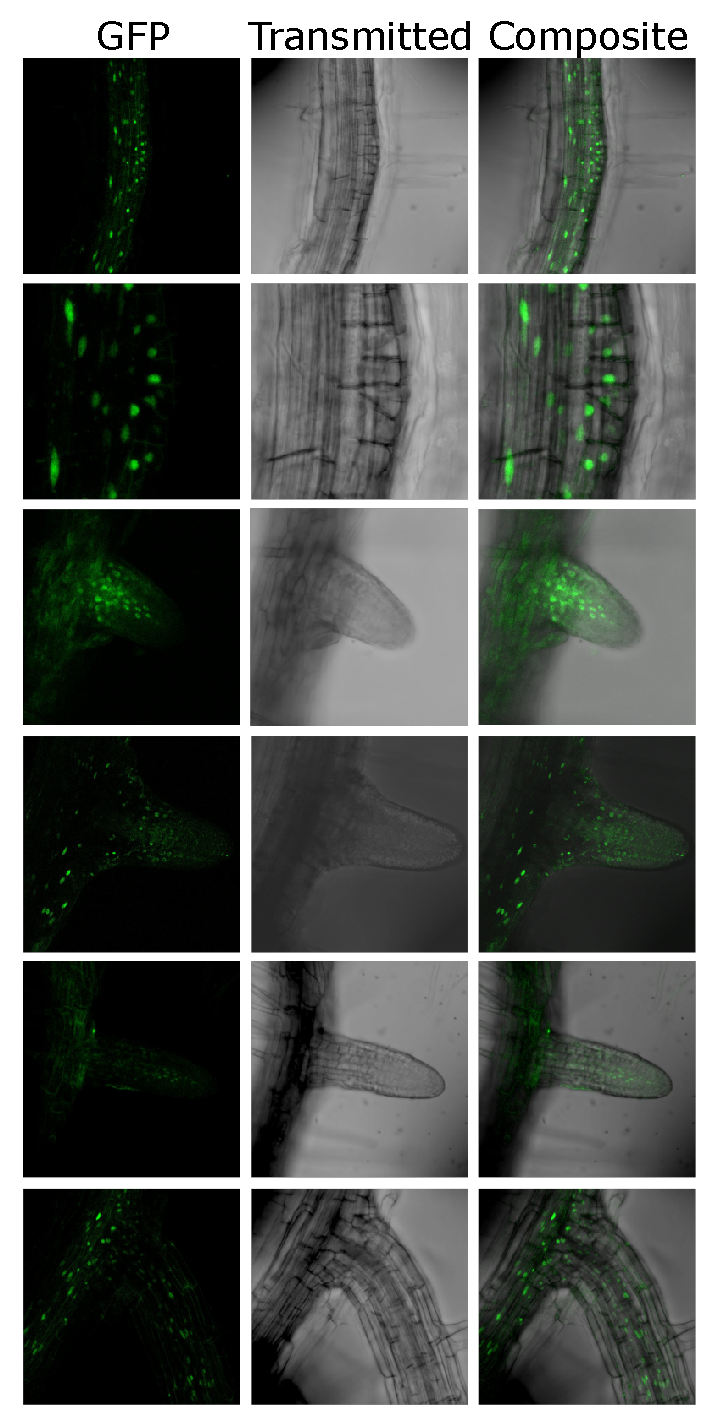
**
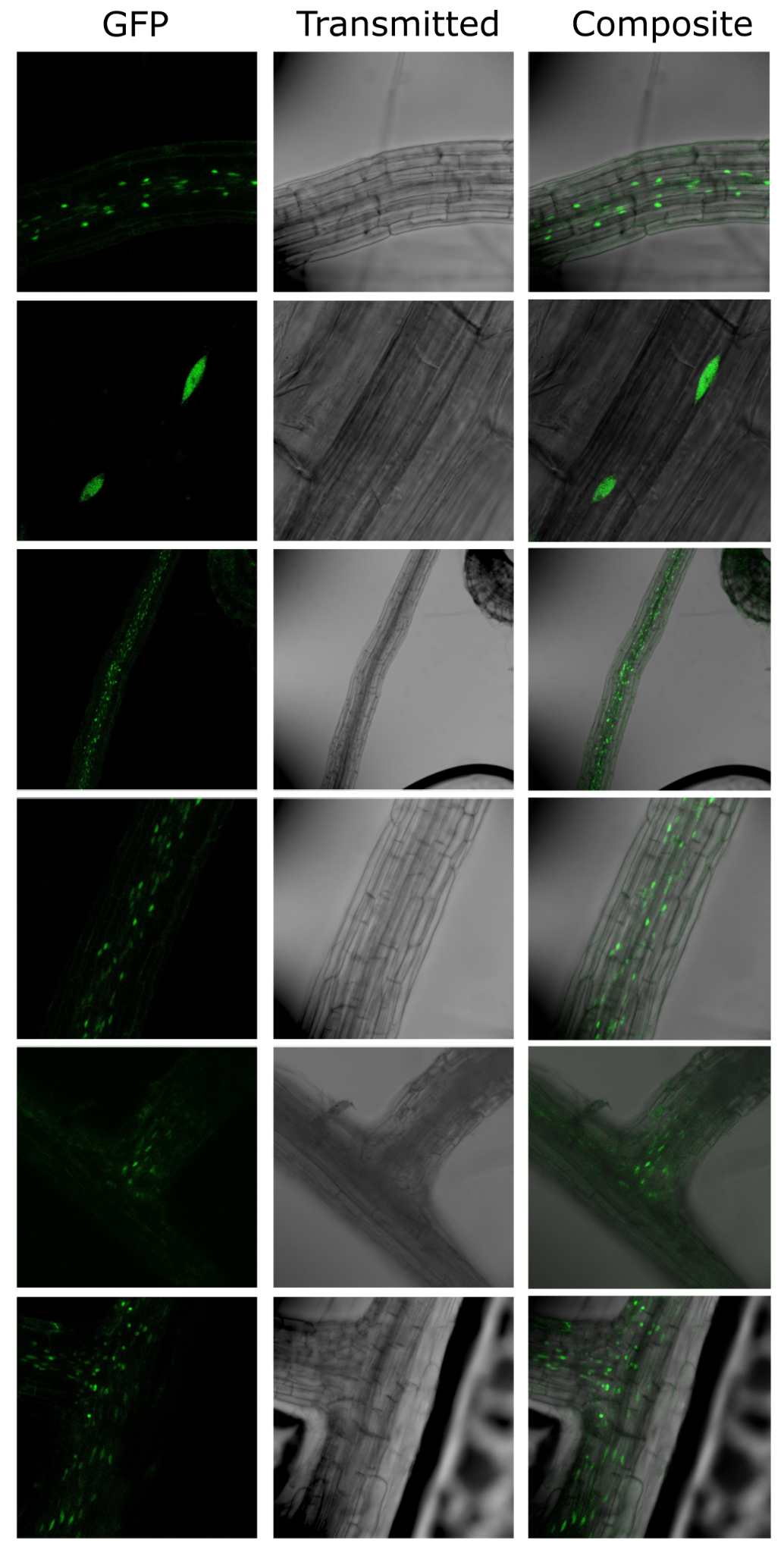
**

**Figure S3.** **Localization of NF-YA10-GFP during lateral root development.**

The expression pattern of NF-YA10 fused to GFP was observed at various stages of lateral root development in 8-day-old pNF-YA10:GFP-NF-YAmiRres (line 1) plants.

**
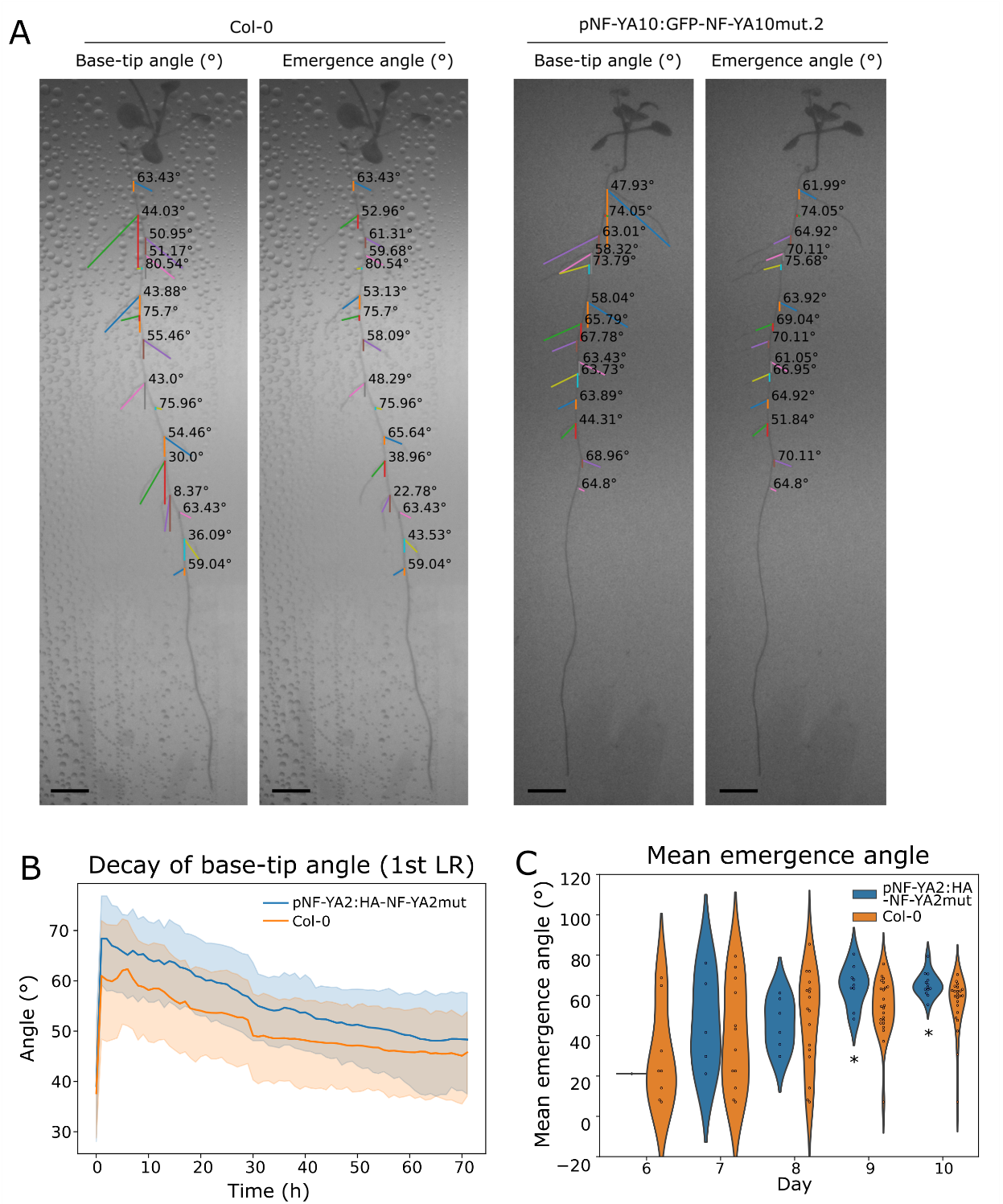
**

**Figure S4. Analysis of novel lateral root curvature parameters in NF-YA2miRres and NF-YA10miRres seedlings using ChronoRoot.** (A) measurements of base-tip and emergence angles. Scale bar = 10 mm. The base-tip angle comparison between lines (as shown in Figure 3D and S4B) was calculated for the first emerged lateral root only. (B) Dynamics of tip decay over time of the first lateral root to emerge along the time of NF-YA2 miRres plants and Col-0.  (C) Mean emergence angle of NF-YA2 miRres and Col-0 roots at different plant ages. For both genotypes, statistical analysis was performed on n=24 (from day 7) for Col-0 and n=13 for NF-YA2 miRres using the Mann-Whitney test. Asterisks indicate a statistical significance p<0.05.

**
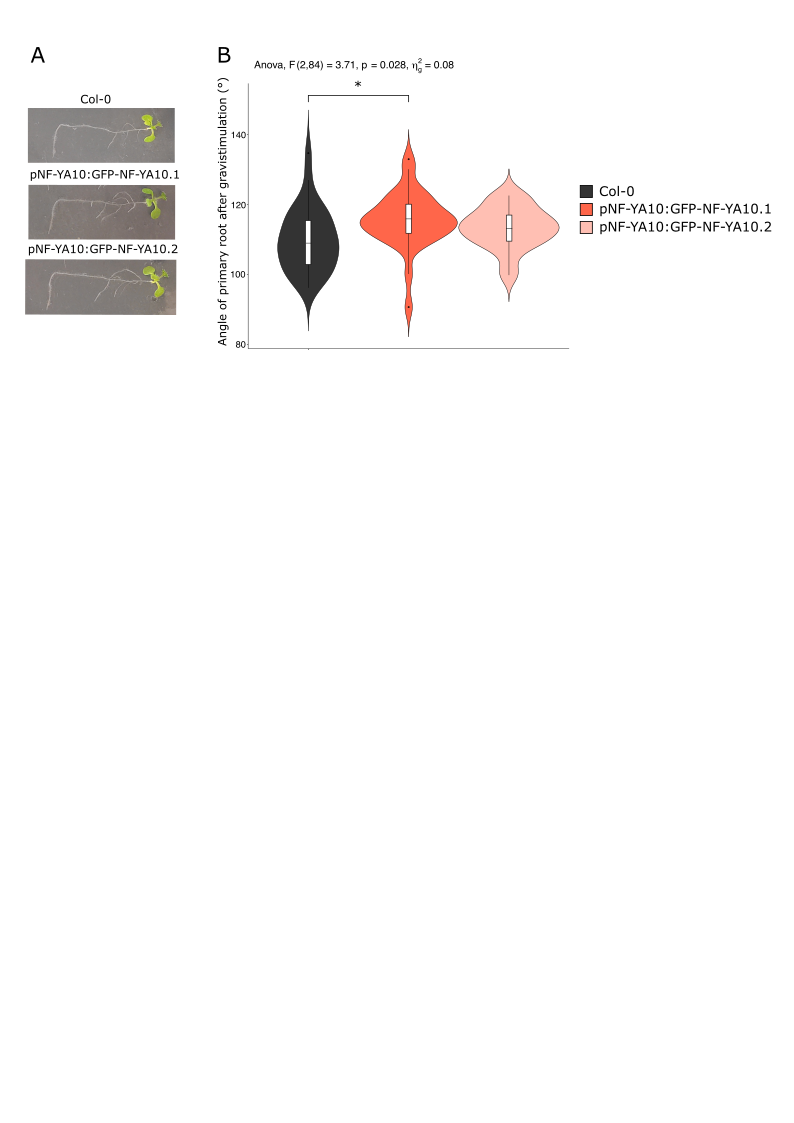
Figure S5. Characterization of the gravitropic response of the main roots of NF-YA10.miRres seedlings.** (A) Representative photographs of 8-day-old NF-YA10.miRres and Col-0 seedlings and (B) mean angle of corresponding primary roots 48 hours after gravistimulation. For both genotypes, statistical analysis was performed on n=29 plants using the Tukey HSD test.


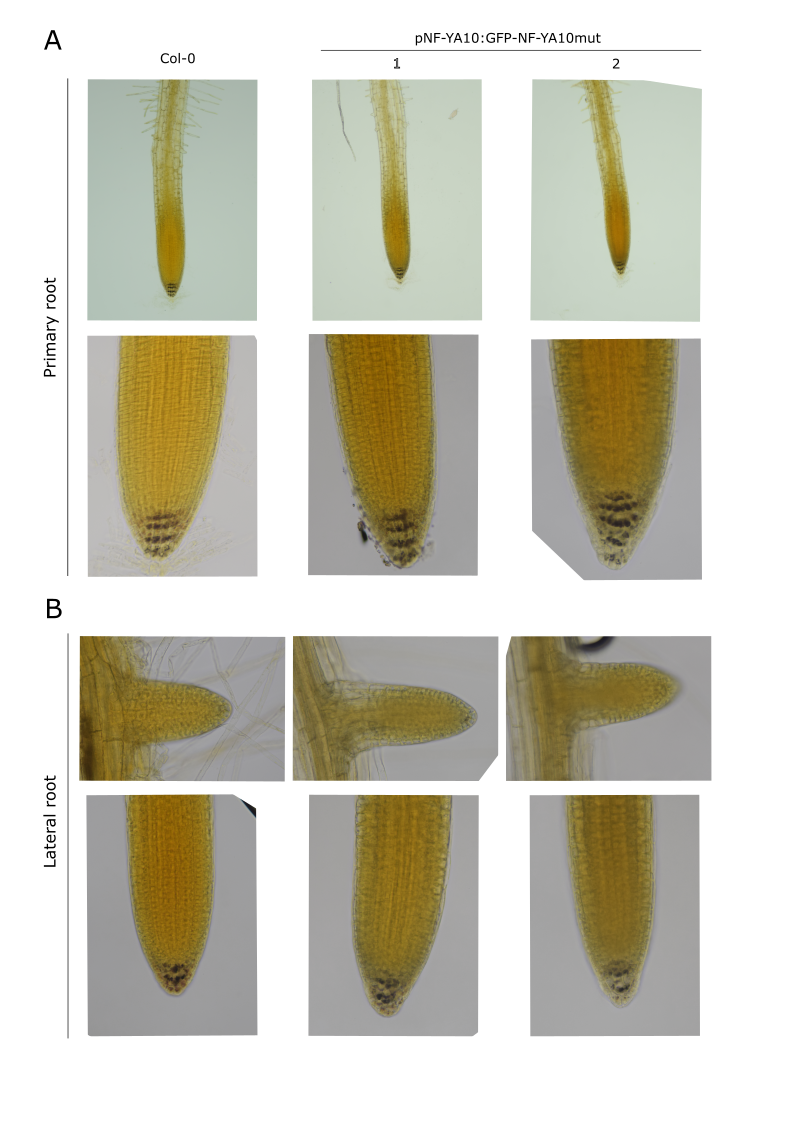


**Figure S6.** **Characterization of amyloplasts main and lateral root tips of NF-YA10 miRres seedlings.** Representative photographs of (A) primary and (B) lateral root apices of 8-day-old NF-YA10 miRres and Col-0 seedlings, showing amyloplasts stained with Lugol.


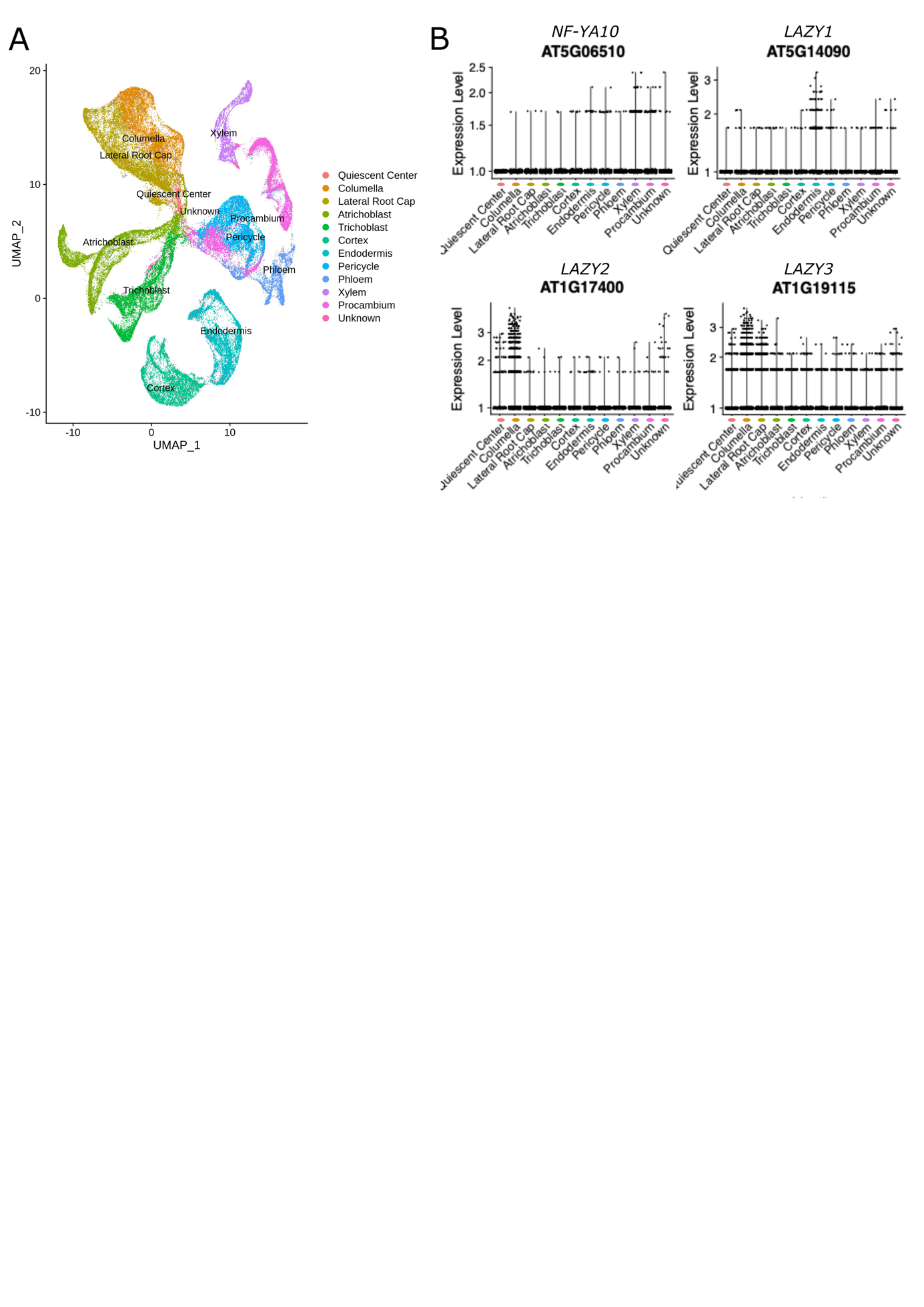


**Figure S7. Expression of *NF-YA10* and *LAZY* genes across multiple cell types in *Arabidopsis thaliana* roots based on single-cell transcriptomics.** (A) UMAP visualization with cell-type labels, using the data from Shahan *et al.*, 2022. The overlap of cell types (e.g., procambium and pericycle) is a result of 2D projection. (B) Violin plots showing the number of cells expressing *NF-YA10*, *LAZY1*, *LAZY2* and *LAZY3* and the corresponding expression levels.
